# 2,4-Dinitro-1-naphthyl 4-toluene­sulfonate

**DOI:** 10.1107/S1600536808010052

**Published:** 2008-04-18

**Authors:** G. Ramachandran, Charles Christopher Kanakam, V. Manivannan

**Affiliations:** a Department of Chemistry, Valliammai Engineering College, Chennai, India; b Department of Physics, Presidency College, Chennai 600 005, India

## Abstract

In the title compound, C_17_H_12_N_2_O_7_S, the dihedral angle between the benzene ring and the naphthyl plane is 26.34 (6)°. The nitro groups make dihedral angles of 40.09 (4) and 37.05 (3)° with the naphthyl plane. In the crystal structure, weak intra- and inter­molecular C—H⋯O inter­actions are observed.

## Related literature

For biological activity, see: Yachi *et al.* (1989 ▶). For the structure of closely related compounds, see: Manivannan *et al.* (2005*a* ▶,*b* ▶). 
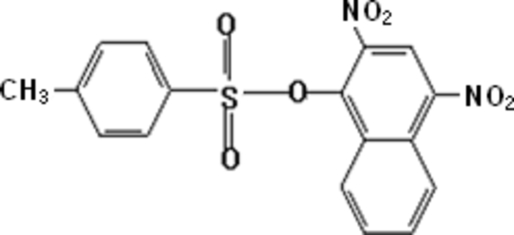


## Experimental

### 

#### Crystal data

C_17_H_12_N_2_O_7_S
*M* *_r_* = 388.35
Monoclinic, 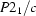

*a* = 13.071 (2) Å
*b* = 7.8660 (13) Å
*c* = 16.595 (3) Å
β = 90.757 (3)°
*V* = 1706.0 (5) Å^3^
*Z* = 4
Mo *K*α radiation
μ = 0.23 mm^−1^
*T* = 295 (2) K
0.36 × 0.25 × 0.13 mm

#### Data collection

Bruker Kappa APEXII diffractometer
Absorption correction: multi-scan (**SADABS**; Sheldrick, 1996 ▶) *T*_min_ = 0.920, *T*_max_ = 0.970
12222 measured reflections
3116 independent reflections
2291 reflections with *I* > 2σ(*I*)
*R*_int_ = 0.031

#### Refinement

*R*[*F* ^2^ > 2σ(*F* ^2^)] = 0.044
*wR*(*F* ^2^) = 0.104
*S* = 1.02
3116 reflections
245 parameters
H-atom parameters constrained
Δρ_max_ = 0.20 e Å^−3^
Δρ_min_ = −0.23 e Å^−3^

### 

Data collection: *APEX2* (Bruker, 2004 ▶); cell refinement: *APEX2*; data reduction: *APEX2*; program(s) used to solve structure: *SHELXS97* (Sheldrick, 2008 ▶); program(s) used to refine structure: *SHELXL97* (Sheldrick, 2008 ▶); molecular graphics: *PLATON* (Spek, 2003 ▶); software used to prepare material for publication: *SHELXL97*.

## Supplementary Material

Crystal structure: contains datablocks I, global. DOI: 10.1107/S1600536808010052/is2287sup1.cif

Structure factors: contains datablocks I. DOI: 10.1107/S1600536808010052/is2287Isup2.hkl

Additional supplementary materials:  crystallographic information; 3D view; checkCIF report

## Figures and Tables

**Table 1:** Hydrogen-bond geometry (Å, °)

*D*—H⋯*A*	*D*—H	H⋯*A*	*D*⋯*A*	*D*—H⋯*A*
C2—H2⋯O5	0.93	2.55	3.194 (3)	127
C14—H14⋯O7	0.93	2.33	2.895 (3)	119
C17—H17⋯O3	0.93	2.48	2.798 (3)	100
C10—H10⋯O1^i^	0.93	2.45	3.327 (3)	157

Symmetry code: (i) 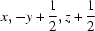.

**Fig. 1.**
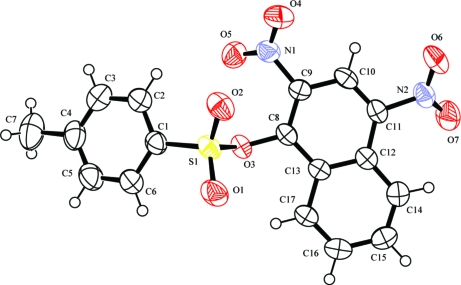
The molecular structure of (I), with atom labels and 50% probability displacement ellipsoids for non-H atoms.

**Fig. 2.**
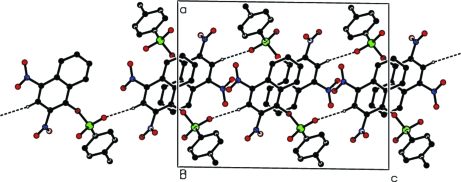
The packing of (I), viewed down the b axis. Hydrogen bonds are shown as dashed lines. H atoms not involved in hydrogen bonding have been omitted.

## supplementary crystallographic information

### Comment

Aromatic sulfonates are used in monitoring the merging of lipids (Yachi *et al.*, 1989). The geometric parameters in the title compound agree with the reported values of similar structures (Manivannan *et al.*, 2005*a*,*b*). The dihedral angle between the mean planes of phenyl and naphthyl rings is 26.34 (6)°. The planes N1/O5/O4 and N2/O6/O7 make the dihedral angles of 40.09 (4) and 37.05 (3)°, respectively, with the naphthyl ring. The torsion angles [O1—S1—C1—C6 = -17.0 (2)° and O2—S1—C1—C2 = 27.9 (2)°] indicate a *syn* conformation of the sulfonyl moiety.

In addition to this, because of the presence of highly electron attracting nitro groups, there are strong dipole-dipole attractions between different molecules in the lattice arrangement. The nitro group substituted naphthylring, which is electron deficient is found to be lying over the electron rich tolyl benzene ring of another molecule in the lattice. This leads to a sort of charge transfer complex.

The enhanced stability of this compound and larger stability of the lattice when compared to other sulfonates reported already, is supported by thermoanalytic studies. This compound is having higher density, melting point and higher lattice energy when compared to others. Another interesting property of this compound is that it possesses antibacterial activity almost equivalent to those of antibiotics. This is attributed to the elongation of the S—O (S1—O3) bond in –S—O–naphthyl ring such that the dissociation to naphthoxy moiety is facilitated. The facile formation of the naphthoxy radical is further supported by the high intensity peak for this specy in the Mass spectra. Kinetic studies also indicate that the rate of hydrolysis (rate of cleavage of the –S—O– bond) is very high when compared to other toluene sulfonates reported already.

The molecular structure is stabilized by weak intramolecular C—H···O interactions and the crystal packing of (I) (Fig. 2) is stabilized by weak intermolecular C—H···O interactions.

### Experimental

Calculated quantity of (10 mmol) of alpha naphthol was dissolved in hot con. sulfuric acid (10 ml) and heated for 10 minutes over a water bath to get disulfonic acid. To this was added (10 ml) of fuming nitric acid in small quantity at a time with stirring. After the addition was over the reaction mixture was kept aside for an hour. It was poured into crushed ice with stirring. The precipitate was filtered, washed with cold water, dried and recrystallized from rectified spirit.

A solution of the above 2,4-dinitronaphthol and triethylamine in acetone was treated with sulfonyl chloride in acetone. This was left as such overnight. The solvent was evaporated and the residue was washed with triethylamine solution. The crude product was recrystallized from ethanol to get diffraction quality crystal of 2,4-dintro-1-naphthyl-4-toluene sulfonate.

### Refinement

H atoms were positioned geometrically and refined using riding model with C—H = 0.93 Å and *U*_iso_(H) = 1.2*U*_eq_(C) for aromatic C—H, and with C—H = 0.96 Å and *U*_iso_(H) = 1.5*U*_eq_(C) for CH_3_.

### Crystal data

**Table:**

C_17_H_12_N_2_O_7_S	*F*_000_ = 800
*M**_r_* = 388.35	*D*_x_ = 1.512 Mg m^−^^3^
Monoclinic, *P*2_1_/*c*	Mo *K*α radiation λ = 0.71073 Å
Hall symbol: -P 2ybc	Cell parameters from 4023 reflections
*a* = 13.071 (2) Å	θ = 1.8–25.2º
*b* = 7.8660 (13) Å	µ = 0.24 mm^−^^1^
*c* = 16.595 (3) Å	*T* = 295 (2) K
β = 90.757 (3)º	Block, colourless
*V* = 1706.0 (5) Å^3^	0.36 × 0.25 × 0.13 mm
*Z* = 4	

### Data collection

**Table:**

Bruker Kappa APEXII diffractometer	3116 independent reflections
Radiation source: fine-focus sealed tube	2291 reflections with *I* > 2σ(*I*)
Monochromator: graphite	*R*_int_ = 0.031
*T* = 295(2) K	θ_max_ = 25.4º
ω and φ scans	θ_min_ = 2.5º
Absorption correction: multi-scan(*SADABS*; Sheldrick, 1996)	*h* = −15→15
*T*_min_ = 0.920, *T*_max_ = 0.970	*k* = −9→9
12222 measured reflections	*l* = −19→19

### Refinement

**Table:**

Refinement on *F*^2^	Secondary atom site location: difference Fourier map
Least-squares matrix: full	Hydrogen site location: inferred from neighbouring sites
*R*[*F*^2^ > 2σ(*F*^2^)] = 0.044	H-atom parameters constrained
*wR*(*F*^2^) = 0.104	*w* = 1/[σ^2^(*F*_o_^2^) + (0.0486*P*)^2^ + 0.4765*P*] where *P* = (*F*_o_^2^ + 2*F*_c_^2^)/3
*S* = 1.02	(Δ/σ)_max_ < 0.001
3116 reflections	Δρ_max_ = 0.20 e Å^−^^3^
245 parameters	Δρ_min_ = −0.23 e Å^−^^3^
Primary atom site location: structure-invariant direct methods	Extinction correction: none

### Fractional atomic coordinates and isotropic or equivalent isotropic displacement parameters (Å^2^)

**Table:**

	*x*	*y*	*z*	*U*_iso_*/*U*_eq_	
S1	0.75961 (5)	0.14928 (8)	0.91732 (3)	0.04636 (19)	
N2	0.47400 (18)	0.4441 (3)	1.21402 (12)	0.0509 (5)	
N1	0.77051 (15)	0.1383 (3)	1.12836 (11)	0.0511 (5)	
C8	0.62399 (17)	0.1694 (3)	1.03092 (12)	0.0379 (5)	
C9	0.66905 (17)	0.2018 (3)	1.10432 (13)	0.0396 (5)	
C10	0.61838 (18)	0.2985 (3)	1.16206 (13)	0.0418 (5)	
H10	0.6505	0.3244	1.2109	0.050*	
C11	0.52268 (18)	0.3540 (3)	1.14659 (12)	0.0394 (5)	
C12	0.47017 (17)	0.3258 (3)	1.07210 (12)	0.0383 (5)	
C13	0.52516 (16)	0.2336 (3)	1.01249 (12)	0.0366 (5)	
C14	0.37180 (18)	0.3875 (3)	1.05167 (14)	0.0462 (6)	
H14	0.3347	0.4476	1.0897	0.055*	
C15	0.33082 (18)	0.3603 (3)	0.97732 (15)	0.0502 (6)	
H15	0.2658	0.4016	0.9651	0.060*	
C16	0.38494 (19)	0.2709 (3)	0.91879 (15)	0.0494 (6)	
H16	0.3558	0.2542	0.8680	0.059*	
C17	0.47966 (18)	0.2082 (3)	0.93546 (13)	0.0421 (5)	
H17	0.5148	0.1484	0.8962	0.051*	
C1	0.82025 (17)	−0.0380 (3)	0.88993 (14)	0.0467 (6)	
C4	0.9179 (2)	−0.3362 (4)	0.84598 (18)	0.0639 (7)	
C2	0.8941 (2)	−0.1075 (4)	0.94025 (17)	0.0660 (8)	
H2	0.9113	−0.0548	0.9888	0.079*	
C3	0.9421 (2)	−0.2557 (4)	0.91778 (19)	0.0744 (9)	
H3	0.9918	−0.3026	0.9516	0.089*	
C6	0.7943 (2)	−0.1167 (4)	0.81845 (15)	0.0583 (7)	
H6	0.7439	−0.0708	0.7848	0.070*	
C5	0.8437 (2)	−0.2639 (4)	0.79741 (18)	0.0671 (8)	
H5	0.8264	−0.3163	0.7489	0.081*	
C7	0.9719 (2)	−0.4974 (4)	0.8216 (2)	0.0947 (11)	
H7A	1.0222	−0.4715	0.7818	0.142*	
H7B	0.9230	−0.5762	0.7995	0.142*	
H7C	1.0049	−0.5470	0.8680	0.142*	
O1	0.70983 (14)	0.2246 (2)	0.84979 (9)	0.0613 (5)	
O2	0.82266 (13)	0.2523 (2)	0.96766 (10)	0.0584 (5)	
O3	0.67103 (11)	0.06829 (18)	0.97377 (8)	0.0431 (4)	
O4	0.82359 (15)	0.2326 (3)	1.16921 (12)	0.0810 (6)	
O5	0.79512 (14)	−0.0046 (3)	1.10830 (11)	0.0644 (5)	
O6	0.53013 (16)	0.5300 (3)	1.25734 (11)	0.0738 (6)	
O7	0.38300 (16)	0.4246 (3)	1.22514 (11)	0.0713 (6)	

### Atomic displacement parameters (Å^2^)

**Table:**

	*U*^11^	*U*^22^	*U*^33^	*U*^12^	*U*^13^	*U*^23^
S1	0.0527 (4)	0.0503 (4)	0.0362 (3)	−0.0065 (3)	0.0072 (3)	−0.0017 (3)
N2	0.0698 (15)	0.0451 (12)	0.0382 (11)	0.0054 (11)	0.0093 (11)	0.0005 (9)
N1	0.0516 (13)	0.0644 (15)	0.0372 (11)	−0.0010 (12)	−0.0001 (9)	0.0005 (11)
C8	0.0473 (13)	0.0341 (12)	0.0324 (11)	−0.0068 (10)	0.0055 (10)	0.0000 (9)
C9	0.0447 (13)	0.0384 (12)	0.0358 (12)	−0.0044 (10)	0.0001 (10)	0.0041 (10)
C10	0.0582 (15)	0.0389 (12)	0.0285 (11)	−0.0081 (11)	0.0000 (10)	0.0002 (10)
C11	0.0532 (14)	0.0313 (11)	0.0338 (11)	−0.0022 (10)	0.0087 (10)	0.0017 (9)
C12	0.0485 (13)	0.0300 (11)	0.0364 (12)	−0.0069 (10)	0.0043 (10)	0.0048 (9)
C13	0.0446 (13)	0.0309 (11)	0.0344 (11)	−0.0090 (10)	0.0013 (10)	0.0028 (9)
C14	0.0521 (14)	0.0383 (13)	0.0485 (14)	−0.0017 (11)	0.0078 (11)	0.0060 (11)
C15	0.0448 (14)	0.0490 (14)	0.0566 (15)	−0.0038 (11)	−0.0045 (12)	0.0106 (13)
C16	0.0564 (15)	0.0488 (14)	0.0427 (13)	−0.0126 (12)	−0.0088 (12)	0.0061 (11)
C17	0.0517 (14)	0.0394 (12)	0.0352 (12)	−0.0088 (11)	0.0001 (10)	0.0011 (10)
C1	0.0405 (13)	0.0579 (15)	0.0420 (13)	−0.0071 (11)	0.0076 (11)	−0.0057 (11)
C4	0.0484 (15)	0.0653 (18)	0.078 (2)	−0.0063 (14)	0.0158 (14)	−0.0175 (16)
C2	0.0531 (16)	0.087 (2)	0.0578 (16)	0.0052 (15)	−0.0036 (13)	−0.0213 (15)
C3	0.0555 (17)	0.089 (2)	0.079 (2)	0.0164 (16)	−0.0041 (15)	−0.0103 (18)
C6	0.0564 (16)	0.0690 (18)	0.0496 (15)	0.0026 (14)	0.0009 (12)	−0.0111 (13)
C5	0.0612 (17)	0.076 (2)	0.0641 (18)	−0.0081 (15)	0.0040 (14)	−0.0264 (16)
C7	0.075 (2)	0.077 (2)	0.133 (3)	0.0067 (18)	0.022 (2)	−0.028 (2)
O1	0.0794 (12)	0.0660 (12)	0.0387 (9)	0.0039 (9)	0.0059 (9)	0.0086 (8)
O2	0.0630 (11)	0.0584 (11)	0.0539 (10)	−0.0189 (9)	0.0080 (8)	−0.0100 (9)
O3	0.0514 (9)	0.0404 (9)	0.0377 (8)	−0.0031 (7)	0.0076 (7)	−0.0054 (7)
O4	0.0617 (12)	0.1075 (17)	0.0734 (13)	−0.0049 (11)	−0.0180 (11)	−0.0267 (12)
O5	0.0660 (12)	0.0632 (12)	0.0640 (12)	0.0149 (10)	−0.0005 (9)	0.0042 (10)
O6	0.0898 (15)	0.0723 (13)	0.0593 (12)	0.0021 (11)	0.0009 (11)	−0.0308 (10)
O7	0.0664 (13)	0.0885 (15)	0.0596 (12)	0.0031 (11)	0.0229 (10)	−0.0065 (10)

### Geometric parameters (Å, °)

**Table:**

S1—O1	1.4179 (17)	C14—H14	0.9300
S1—O2	1.4196 (16)	C15—C16	1.399 (3)
S1—O3	1.6287 (16)	C15—H15	0.9300
S1—C1	1.736 (3)	C16—C17	1.358 (3)
N2—O7	1.216 (3)	C16—H16	0.9300
N2—O6	1.224 (3)	C17—H17	0.9300
N2—C11	1.476 (3)	C1—C6	1.377 (3)
N1—O4	1.216 (3)	C1—C2	1.381 (3)
N1—O5	1.216 (3)	C4—C5	1.376 (4)
N1—C9	1.467 (3)	C4—C3	1.382 (4)
C8—C9	1.370 (3)	C4—C7	1.509 (4)
C8—O3	1.388 (2)	C2—C3	1.378 (4)
C8—C13	1.416 (3)	C2—H2	0.9300
C9—C10	1.397 (3)	C3—H3	0.9300
C10—C11	1.346 (3)	C6—C5	1.373 (4)
C10—H10	0.9300	C6—H6	0.9300
C11—C12	1.423 (3)	C5—H5	0.9300
C12—C14	1.411 (3)	C7—H7A	0.9600
C12—C13	1.429 (3)	C7—H7B	0.9600
C13—C17	1.417 (3)	C7—H7C	0.9600
C14—C15	1.356 (3)		
			
O1—S1—O2	118.91 (11)	C14—C15—H15	119.5
O1—S1—O3	107.20 (10)	C16—C15—H15	119.5
O2—S1—O3	107.24 (9)	C17—C16—C15	120.6 (2)
O1—S1—C1	110.71 (11)	C17—C16—H16	119.7
O2—S1—C1	112.01 (11)	C15—C16—H16	119.7
O3—S1—C1	98.58 (10)	C16—C17—C13	120.2 (2)
O7—N2—O6	124.1 (2)	C16—C17—H17	119.9
O7—N2—C11	119.1 (2)	C13—C17—H17	119.9
O6—N2—C11	116.7 (2)	C6—C1—C2	120.4 (2)
O4—N1—O5	124.4 (2)	C6—C1—S1	119.9 (2)
O4—N1—C9	116.8 (2)	C2—C1—S1	119.70 (19)
O5—N1—C9	118.8 (2)	C5—C4—C3	117.8 (3)
C9—C8—O3	121.7 (2)	C5—C4—C7	121.3 (3)
C9—C8—C13	120.4 (2)	C3—C4—C7	120.9 (3)
O3—C8—C13	117.89 (18)	C3—C2—C1	119.2 (3)
C8—C9—C10	120.6 (2)	C3—C2—H2	120.4
C8—C9—N1	123.7 (2)	C1—C2—H2	120.4
C10—C9—N1	115.71 (19)	C2—C3—C4	121.4 (3)
C11—C10—C9	119.6 (2)	C2—C3—H3	119.3
C11—C10—H10	120.2	C4—C3—H3	119.3
C9—C10—H10	120.2	C5—C6—C1	119.2 (3)
C10—C11—C12	123.4 (2)	C5—C6—H6	120.4
C10—C11—N2	114.8 (2)	C1—C6—H6	120.4
C12—C11—N2	121.7 (2)	C6—C5—C4	122.0 (3)
C14—C12—C11	125.6 (2)	C6—C5—H5	119.0
C14—C12—C13	118.3 (2)	C4—C5—H5	119.0
C11—C12—C13	116.1 (2)	C4—C7—H7A	109.5
C8—C13—C17	121.0 (2)	C4—C7—H7B	109.5
C8—C13—C12	119.83 (19)	H7A—C7—H7B	109.5
C17—C13—C12	119.1 (2)	C4—C7—H7C	109.5
C15—C14—C12	120.8 (2)	H7A—C7—H7C	109.5
C15—C14—H14	119.6	H7B—C7—H7C	109.5
C12—C14—H14	119.6	C8—O3—S1	119.58 (13)
C14—C15—C16	120.9 (2)		
			
O3—C8—C9—C10	−177.46 (19)	C11—C12—C14—C15	176.7 (2)
C13—C8—C9—C10	0.0 (3)	C13—C12—C14—C15	−0.2 (3)
O3—C8—C9—N1	1.8 (3)	C12—C14—C15—C16	−0.2 (3)
C13—C8—C9—N1	179.21 (19)	C14—C15—C16—C17	0.5 (3)
O4—N1—C9—C8	143.2 (2)	C15—C16—C17—C13	−0.3 (3)
O5—N1—C9—C8	−38.6 (3)	C8—C13—C17—C16	−179.9 (2)
O4—N1—C9—C10	−37.5 (3)	C12—C13—C17—C16	−0.1 (3)
O5—N1—C9—C10	140.7 (2)	O1—S1—C1—C6	−17.0 (2)
C8—C9—C10—C11	2.9 (3)	O2—S1—C1—C6	−152.30 (19)
N1—C9—C10—C11	−176.4 (2)	O3—S1—C1—C6	95.1 (2)
C9—C10—C11—C12	−2.8 (3)	O1—S1—C1—C2	163.2 (2)
C9—C10—C11—N2	175.77 (19)	O2—S1—C1—C2	27.9 (2)
O7—N2—C11—C10	−143.1 (2)	O3—S1—C1—C2	−84.7 (2)
O6—N2—C11—C10	34.3 (3)	C6—C1—C2—C3	0.5 (4)
O7—N2—C11—C12	35.5 (3)	S1—C1—C2—C3	−179.7 (2)
O6—N2—C11—C12	−147.1 (2)	C1—C2—C3—C4	0.0 (5)
C10—C11—C12—C14	−177.1 (2)	C5—C4—C3—C2	−0.3 (4)
N2—C11—C12—C14	4.5 (3)	C7—C4—C3—C2	179.2 (3)
C10—C11—C12—C13	−0.1 (3)	C2—C1—C6—C5	−0.8 (4)
N2—C11—C12—C13	−178.59 (19)	S1—C1—C6—C5	179.4 (2)
C9—C8—C13—C17	176.87 (19)	C1—C6—C5—C4	0.6 (4)
O3—C8—C13—C17	−5.6 (3)	C3—C4—C5—C6	0.0 (4)
C9—C8—C13—C12	−2.9 (3)	C7—C4—C5—C6	−179.5 (3)
O3—C8—C13—C12	174.60 (17)	C9—C8—O3—S1	−80.6 (2)
C14—C12—C13—C8	−179.86 (19)	C13—C8—O3—S1	101.93 (19)
C11—C12—C13—C8	3.0 (3)	O1—S1—O3—C8	−86.68 (17)
C14—C12—C13—C17	0.3 (3)	O2—S1—O3—C8	42.07 (17)
C11—C12—C13—C17	−176.83 (19)	C1—S1—O3—C8	158.41 (16)

### Hydrogen-bond geometry (Å, °)

**Table:**

*D*—H···*A*	*D*—H	H···*A*	*D*···*A*	*D*—H···*A*
C2—H2···O5	0.93	2.55	3.194 (3)	127
C14—H14···O7	0.93	2.33	2.895 (3)	119
C17—H17···O3	0.93	2.48	2.798 (3)	100
C10—H10···O1^i^	0.93	2.45	3.327 (3)	157

Symmetry codes: (i) *x*, −*y*+1/2, *z*+1/2.
